# Application of Ultrasound-Assisted Extraction for Isolating Rhamnetin from Clove (*Syzygium aromaticum*) Flower Buds: Process Optimization, Identification, and Bioactivity Assessment

**DOI:** 10.3390/molecules31213857

**Published:** 2026-10-26

**Authors:** Marek Pieszka, Joanna Romanek, Władysław Migdał, Augustyn Roch

**Affiliations:** 1 Department of Animal Nutrition and Feed Sciences, National Research Institute of Animal Production, Krakowska Str. 1, 32-083 Balice, Poland; joanna.romanek@iz.edu.pl; 2 Department of Animal Products Technology, Faculty of Food Technology, University of Agriculture in Kraków, ul. Balicka 122, 31-149 Krakow, Poland; wladyslaw.migdal@urk.edu.pl; 3 Agro Food S.A., ul. Żarnowiecka 56, 42-436 Pilica, Poland; august1roch@gmail.com

**Keywords:** *Syzygium aromaticum*, ultrasound-assisted extraction (UAE), green chemistry, rhamnetin, antioxidant activity, enterocytes, cytotoxicity, transepithelial electrical resistance (TEER)

## Abstract

The present study aimed to evaluate the effect of ultrasound-assisted extraction (UAE) parameters, specifically extraction time, temperature, and ultrasound amplitude, on extraction yield, bioactive compound content, and antioxidant and cytotoxic activities of ethanolic extracts obtained from flower buds of the clove plant (*Syzygium aromaticum*). Extraction was performed using 70% ethanol as an environmentally friendly solvent, and the sonication conditions were systematically optimized with clear statistical justification through experimental design. The total phenolic content (TPC) was estimated spectrophotometrically, and the qualitative and quantitative profiles of flavonoids (rhamnetin and isorhamnetin) were analyzed by reversed-phase ultra-high-performance liquid chromatography coupled with electrospray ionization mass spectrometry. Rhamnetin and isorhamnetin were selected as specific analytical markers due to their high stability, unique methoxy-substitution patterns, and pivotal role in mediating biological efficacy. DPPH, ABTS, and FRAP assays were conducted to assess the antioxidant properties of the extracts, incorporating pure individual reference standards (gallic acid, rhamnetin, and isorhamnetin) at matching concentrations to account for potential synergistic or antagonistic multi-component interactions. Furthermore, the extract obtained under the optimized extraction conditions was subjected to in vitro evaluation using porcine enterocyte cell lines. The cytotoxic properties of the extract and its effects on cell viability, reactive oxygen species (ROS) generation, and epithelial barrier integrity were assessed, with the latter determined by measuring transepithelial electrical resistance (TEER). UAE parameters significantly influenced extraction efficiency. The optimal extraction conditions, i.e., extraction time: 20 min; temperature: −30 °C; amplitude: 90 µm; power density: 30 W/mL, provided the highest extraction yield (21.80%), which was significantly greater than those obtained using conventional extraction methods (maceration and hydrodistillation). The obtained extract exhibited the highest TPC (320.08 mg gallic acid equivalent/100 g dry matter [DM]) and the highest concentrations of rhamnetin (241.03 ppm) and isorhamnetin (32.80 ppm). Consequently, it showed excellent antioxidant activity, with DPPH radical scavenging activity of 97.28%, FRAP value of 466.27 mg Trolox/100 g DM, and ABTS value of 12.98 µM Trolox/100 g DM. In cell-based in vitro experiments, the highest extract concentrations (≥1000 mg/L) significantly reduced enterocyte viability (*p* ≤ 0.002), impaired epithelial barrier integrity as determined by TEER (*p* ≤ 0.005), and induced oxidative stress after 24 h of exposure (*p* ≤ 0.001). Interestingly, short-term exposure (6 h) to high extract concentrations was associated with decreased intracellular ROS levels (*p* ≤ 0.05). Lower extract concentrations showed no apparent disruptive effects on intestinal epithelial barrier integrity under the tested conditions. Lower extract concentrations (*p* ≤ 005) were safe and did not disrupt intestinal epithelial barrier integrity. Thus, UAE with ethanol solvent represents a highly efficient and environmentally friendly alternative to conventional methods for extracting bioactive compounds from clove buds. The obtained extract exhibited a distinct dose- and exposure duration-dependent dual effect, ranging from strong antioxidant activity at low concentrations to cytotoxic effects at high concentrations; this finding highlights the potential utility of this extract in pharmaceuticals and functional foods, provided its dose is carefully regulated.

## 1. Introduction

Natural herbal products serve as the cornerstone of modern pharmacognosy. Their therapeutic properties, including antiviral, antioxidant, antidiabetic, and hepatoprotective effects, are primarily attributed to the presence of secondary metabolites. Unlike primary metabolites, secondary metabolites are synthesized by plants in response to environmental stress and serve as defense mechanisms against pathogens and herbivores [1]. Secondary metabolites play a vital role in plant defense, providing protection against pathogens and deterring herbivorous animals through their distinct taste profiles. Since several centuries, many plants have been widely used as culinary spices. Extensive historical observations and scientific studies have demonstrated that certain spices, such as cloves and ginger, may confer beneficial health effects on humans when consumed as dietary components. Among the various secondary metabolites produced by plants, flavonoids—a specific class of polyphenols—play a key role in color and flavor development. To date, more than 8000 different flavonoid structures have been identified, which exhibit unique biological profiles due to differences in their functional moieties [2,3]. Flavonoids are a class of secondary metabolites responsible for color and taste and exhibit a wide range of biological and pharmacological effects [4]. Flavonoids primarily contains a chromone group (A and C) substituted with a phenyl group (B). A diverse range of flavonoid compounds are present in fruits and vegetables (including more than 8000 identified flavonoid compounds). This number results from variations in the basic structures of their functional moieties, which afford each compound with distinct characteristics. Therefore, flavonoids can be divided into different classes of compounds, among which flavones, flavonols, isoflavones, flavanones, and anthocyanidins are the important ones [2,3]. To establish clear research significance and novelty, ultrasound-assisted extraction (UAE) offers distinct advantages over conventional isolation methodologies (such as Soxhlet extraction, maceration, or standard hydrodistillation) for recovering heat-sensitive flavonols like rhamnetin from clove flower buds. Conventional methods frequently suffer from prolonged thermal exposure, leading to thermal degradation of active constituents, substantial organic solvent consumption, and lower extraction yields. In contrast, UAE utilizes acoustic cavitation phenomena, which mechanically disrupt plant cell walls, enhance mass transfer, and facilitate rapid penetration of green solvents (such as 70% aqueous ethanol) at controlled, low temperatures (−30 °C). This combination effectively prevents thermal decomposition, preserves the structural integrity of rhamnetin, and drastically shortens processing time while maximizing yield.

Rhamnetin (3,5,4′-trihydroxy-7-methoxyflavone; C_16_H_12_O_7_) is a naturally occurring flavonol predominantly identified in its O-glycoside form. The molecular structure is characterized by the chromone skeleton with hydroxyl groups at positions 3, 5, and 4′ and a methoxy group (-OCH_3_) at position 7. Compared to classical quercetin, rhamnetin shows significantly higher metabolic stability and bioavailability due to the presence of the methoxy group at position 7 [5]. The ability of rhamnetin to neutralize reactive oxygen species (ROS) is directly correlated to the number of hydroxyl groups. Rhamnetin, as an effective ROS scavenger, can donate electrons and H^+^ ions, thereby stabilizing free radicals [6].

Rhamnetin is present in many plant species with documented medicinal properties, including herbs and spice plants such as *Ammi visnaga* L., *Thymus nummularius* [7], *Moringa oleifera* L. [8], and *Salvia* L.; in fruits such as grapes (*Vitis vinifera*), oranges, mangoes, fruits of the genus *Prunus*, and jackfruit [9,10]; and in halophytes, where plants such as *Salicornia europaea* accumulate rhamnetin, utilizing it for adapting to high salinity conditions [11]. Rhamnetin acts through multiple mechanisms in combating oxidative stress. Because of the high number of OH groups in its structure, rhamnetin can act as an effective antioxidant by donating up to four electrons and H^+^ ions to ROS, thus stabilizing them; this assumption has been confirmed in DPPH assays, where rhamnetin showed higher efficacy than many other isolated flavonoids [12]. As confirmed previously, the specific structure of rhamnetin (presence of hydroxyl groups in the B ring and the 5-OH group in the A ring) enables its stable binding to key kinase proteins, which contributes to its biological activity [13]. Rhamnetin exhibits strong anti-inflammatory properties by inhibiting pathways such as MAPK (p38, ERK, JNK, NF-κB, and AP-1) and COX-2 [13]. Previous studies have demonstrated the high efficacy of rhamnetin in inhibiting the production of proinflammatory cytokines (e.g., TNF-α and IL-6) and nitric oxide (NO) in lipopolysaccharide (LPS)-stimulated macrophages [14]. Rhamnetin can also chelate and inhibit pro-oxidative enzymes such as xanthine oxidase (XOD) and β-carotene-15,15-dioxygenase [15,16]. In vitro studies on H9c2 myocytes have shown that rhamnetin not only reduces ROS levels but also activates endogenous antioxidant enzymes, protecting the heart against cardiovascular damage [17]. Rhamnetin decreased the concentration of free radicals and increased the expression of antioxidant enzymes in cells. Therefore, using antioxidant flavonoids, such as rhamnetin, could be an effective strategy to prevent oxidative stress-induced heart damage.

A high ROS level poses a serious threat to health; consequently, the occurrence and biological activity of natural antioxidants are being increasingly studied [18]. In living organisms, antioxidants perform several functions, including elimination of free radicals, quenching of singlet oxygen, interference with radical reactions, chelation of metals that catalyze oxidation processes, and inhibition of certain enzymes (e.g., oxidases). Flavonoids participate in all these processes [19].

The antioxidant potential of flavonoid compounds is directly proportional to the amount of OH groups present in their structure. Recent studies have also indicated that rhamnetin can modulate the anti-infective response. For example, rhamnetin isolated from *F. caryophylli* exhibits significant inhibitory activity against influenza viruses [20]. Latest studies have shown that rhamnetin inhibits viral replication by interacting with structural proteins of pathogens and modulating host signaling pathways [14,21]. Although the majority of studies still focus on whole plant extracts, an increasing number of publications identify rhamnetin as the main factor influencing the observed biological activity [21,22].

A promising source of rhamnetin is the buds of the clove tree (*Syzygium aromaticum*), belonging to the Myrtaceae family, which originates from India, Pakistan, and Indonesia [23]. In the clove plant, clove buds contain an abundant amount of dietary phenolic compounds, which have numerous applications in traditional medicine [24]. Studies have shown that clove extracts exhibit antibacterial, antifungal, anti-inflammatory, analgesic, antioxidant, antiglycation, and anti-aging activities [25,26,27]. The primary components of clove extracts are eugenol, isoeugenol, farnesol, and nerolidol as the main aglycones. Clove is also a rich source of rhamnetin.

Clove extracts are mainly obtained using Soxhlet extraction, hydrodistillation, superheated steam extraction, supercritical fluid extraction using CO_2_, microwave-assisted extraction, and ultrasound-assisted extraction (UAE) [28,29,30]. Among these various extraction techniques, UAE using ethyl alcohol as a solvent in a closed loop system is the most efficient and environmentally friendly method.

With the increase in the prevalence of non-communicable diseases (diabetes, cancer, and neurodegenerative disorders) and viral diseases, including SARS-CoV-2 infection, isolated flavonoids such as rhamnetin are a promising avenue for designing new drugs with a high safety profile.

## 2. Materials and Methods

### 2.1. Materials and Extraction of Bioactive Substances from Flower Buds (Eugenia caryophyllata)

The cloves were purchased in 2025 from a supermarket in Krakow, Poland. Before extraction, the cloves were ground on a Fritsch PULVERISETTE 25 laboratory mill (Germany) with a mesh diameter of 1 × 1 mm. The extracts were obtained by sonication under the following conditions. Clove samples weighing 30 g ± 0.01 g were ground, and the samples were then pre-extracted with 270 mL of 70% ethanol at 0 °C to −30 °C. After 30 min, the solution was extracted on a Hielscher Ultrasonics UP200ht sonifier (Germany). The extraction conditions are shown in Table 1. The samples were extracted for 10 or 20 min at 0 °C, −10 °C, and −30 °C using ultrasound waves with a power density and amplitude of 30 W/mL and 90 μm, respectively, using a 14-mm-diameter sonotrode (S26d14). Following extraction, each extract was centrifuged at 5000 *g* for 15 min (Heraeus Biofuge Stratos, Thermo Scientific, Illkirch, France). The resulting extracts were then filtered through a Whatman Grade 1 filter paper. After obtaining the alcoholic extracts, the solvent was evaporated under nitrogen at 30 °C. The obtained extracts were then transferred to sealed dark glass tubes under nitrogen and refrigerated at 4 °C for further chemical analysis. Extraction efficiency was calculated based on the ratio of the extracted mass to the initial leaf mass (dry weight) and expressed as percent.

### 2.2. Reagents

Rhamnetin standard, 2,2-diphenyl-1-picrylhydrazyl (DPPH), 2,2-azobis-(2-amidinopropane) (ABAP), 2,2-azobis(3-ethylbenzothiazoline-6-sulfonic acid) (ABTS), gallic acid, and Trolox were purchased from Merck-Sigma-Aldrich (St. Louis, MO, USA). Ethyl alcohol [96% analytical grade], Na_2_CO_3_, Folin–Ciocalteu reagent, Fe^3+^-TPTZ (ferric-2,4,6-tripyridyl-s-triazine complex), FeSO_4_·7H_2_O, FeCl_3_, HCl-Tris, and acetate buffer (pH 3.6) were obtained from Chempur (Piekary Śląskie, Poland). Deionized water (PURELab Chorus 2 (RO/DI) was obtained from ELGA LabWater, (Lane End, High Wycombe, UK).

### 2.3. Rhamnetin Isolation and Detection by Reversed-Phase Ultra High-Performance Liquid Chromatography Electrospray Ionization Mass Spectrometry (RP-UHPLC-ESI-MS)

Rhamnetin was detected according to the modified method of Biesaga & Pyrzyska [31] and Mildner-Skudlarz et al. [32]. RP-UHPLC-ESI-MS analysis was performed using a C18 column and a Dionex UltiMate 3000 UHPLC system (Thermo Fisher Scientific, Sunnyvale, CA, USA) coupled to a Bruker maXis impact ultrahigh resolution orthogonal quadrupole-time-of-flight accelerator (Bruker Daltonik, Bremen, Germany) equipped with an ESI source and operated in both positive and negative ion modes. RP chromatographic separation was achieved with a Kinetex™ 1.7 µm C18 100 Å, LC column (100 × 2.1 mm, Phenomenex, Torrance, CA, USA) according to the method of Biesaga and Pyrzyńska (2013) [31]. The ESI-MS settings were previously reported by Mildner-Szkudlarz et al. (2015) [32]. The molecular ion [M-H]^-^ for rhamnetin was extracted from full scan chromatograms (±0.005 *m*/*z*), and its peak area was integrated with TASQ 2.1 (Bruker Daltonik, Bremen, Germany). The compounds present in each sample were identified based on retention time of standard and molecular mass and structural information from the MS detector during MS/MS experiments. The limit of quantification (where S/N > 15) determined for rhamnetin was 0.03 ppm. Rhamnetin standard added to the sample at 1 ppm concentration showed a recovery of 98% ± 3%.

### 2.4. Determination of Total Polyphenol Content by the Folin–Ciocalteu Method

The Folin–Ciocalteu method described by Singleton and Rossi (1965) was used to estimate total polyphenol content [33]. The calibration curve was prepared using gallic acid (GA) as a standard (Sigma-Aldrich) based on the analysis of standard solutions in the concentration range of 20–400 mg/L, with R^2^ = 0.999 as the coefficient of determination. For the analysis purpose, 0.5 mL of prepared extract samples, 0.5 mL of Folin–Ciocalteu reagent (Chempur), and 1.5 mL of freshly prepared sodium carbonate (Na_2_CO_3_) solution in water (200 mg/mL) were introduced into a 25 mL volumetric flask and diluted to the final volume with demineralized water. The solutions were mixed and left for 30 min at 20 °C. The absorbance value was measured at 765 nm wavelength for extract samples against a blank sample containing only solvents and reagents by using a Beckmann 640 spectrophotometer (Fullerton, CA, USA). The total phenolic content (TPC) was expressed as mg of gallic acid equivalent (GAE) extracted from 1 g of plant material (mg GAE/g). Each sample was prepared in triplicate. The data are expressed as mean ± standard deviation.

### 2.5. Ferric Reducing Antioxidant Power (FRAP)

The FRAP assay was conducted using the method of Benzie and Strain (1999) [34]. FRAP reagent was prepared by mixing 300 mM acetate buffer (pH 3.6; 3.1 g of sodium acetate trihydrate, 16 mL of glacial acetic acid, and distilled water [to make up the total volume to 1 L]), 10 mM TPTZ (2,4,6-tri (2-pyridyl)-striazine) in 40 mM HCl, and 20 mM FeCl_3_·6H_2_O in the ratio of 10:1:1. Freshly prepared FRAP reagent (1000 μL) was warmed at 37 °C and mixed with 100 μL of samples and standards. The samples were kept for 30 min, and the mixture was subsequently transferred to a plastic microplate. The absorbance value was measured at 595 nm wavelength using the Beckmann DU 640 spectrophotometer. The result was expressed as milligrams of Trolox equivalents per 100 g of sample (mg TE/g of dry weight [DW]). Each sample was prepared in triplicate. Data are expressed as mean ± standard deviation.

### 2.6. Antioxidant Activity Determination by DPPH Radical Scavenging Assay

Antioxidant activity was estimated by DPPH radical scavenging assay according to the methodology developed by Sánchez-Moreno et al. (1998) [35], with modifications by Szlachta and Małecka (2008) [36]. The DPPH radical standard (2,2-diphenyl-1-picrylhydrazyl) was purchased from Sigma-Aldrich. The radical solution undergoes reduction in the presence of an antioxidant, accompanied by a color change in the solution from purple to yellow. The stronger the antioxidant properties of the test sample, the greater is the decrease in absorbance reflecting the reduction of the DPPH radical. Conversely, samples with a lower capacity to scavenge free radicals show a smaller decrease in absorbance. For this assay, 1 mL of the extract was mixed with 5 mL of DPPH solution (0.135 mM) in 80% (*v*/*v*) ethanol, and the mixture was then incubated for 30 min at ambient temperature in the dark to determine the DPPH radical scavenging activity in methanolic extracts from clove buds.

During the analysis, 0.1 mL of the obtained extract was added to 3.9 mL of the radical solution at a concentration of 6 × 10^−5^ mol/dL in 80% (*v*/*v*) ethanol. The mixture was then incubated in the dark for 30 min at ambient temperature and subsequently measured at 5-min intervals for 30 min using a UV-Vis spectrophotometer (Beckmann 640) at 515 nm wavelength. Antioxidant activity was expressed as the percentage of inhibition calculated according to the following equation:

[%] inhibition = [(Ac(0) − Aa(t))/Ac(0)] × 100(1)

where Ac(0): absorbance of the control sample at time t = 0 Aa(t): absorbance of the test sample measured every 5 min.

### 2.7. Antioxidant Activity Determination Using the ABTS Method

The ABTS reagent (2,2-azobis(3-ethylbenzothiazoline-6-sulfonic acid) was used to determine the total antioxidant activity of the test extracts according to the modified method reported by Hwang and Thi (2014) [37]. For the analysis, a 7 mM ABTS solution was prepared using a 2.45 mM potassium persulfate solution as the solvent. The ABTS solution in potassium persulfate was incubated for 24 h at room temperature in the dark. Following incubation, the ABTS solution was diluted until its absorbance value at 734 nm wavelength reached 0.700 ± 0.005. Next, 2500 μL of the ABTS solution in potassium persulfate was added to 25 μL of the test extract (or standard compound solution), and the mixture was incubated for 6 min. Three independent samples were prepared for each test extract. Following incubation, measurements were performed using a Beckmann 640 spectrophotometer at 734 nm wavelength. The obtained values were compared with a calibration curve prepared for Trolox solutions at the following concentrations: 0, 0.1, 0.5, 1, 5, 10, and 50 mM/L. The results were expressed as Trolox equivalents [μM Trolox × g^−1^ of raw material] and RSA [%].

### 2.8. Isolation of Enterocytes and Cell Culture

Enterocytes were retrieved using the protocol of Vermeire et al. (2021) [38] with slight modifications. Immediately after slaughtering, small intestinal tissue was obtained and placed in cold phosphate-buffered saline (PBS) containing 2% antibiotic antimycotic solution (100×) (AAS, Merck). Residual fat and pancreatic tissue were removed, and the sample was rinsed with ice-cold sterile PBS solution. The tissue was then cut into the smallest possible pieces using sterile scissors. The tissue fragments were subsequently transferred to a 15 mL conical tube. Intestinal tissues were then incubated in 35 mL of cold dissociation buffer 1 (30 mM ethylenediaminetetraacetic acid (EDTA, Merck), 1.5 mM dithiothreitol (Merck), and 6 μM Rho kinase (ROCK) inhibitor (Y-27632, Merck) in PBS) for 30 min on ice. At 5-min intervals, the tissues were shaken for 10–15 s. After 30 min, the samples were transferred to 35 mL of warm (37 °C) dissociation buffer 2 (30 mM EDTA and 6 μM Y-27632 in PBS) for 10 min. The tissue was then transferred to cold, sterile PBS and incubated for 5 min, with shaking every 1–2 min [38]. The pellet was filtered through a 70-µm cell strainer (Corning) and resuspended in medium. Cells were cultured in DMEM/F12 medium supplemented with 10% FBS (Merck), 1% ITS (Gibco), 1% AAS (Merck), and 5 ng/mL EGF at 37 °C in 5% CO_2_ and 90% humidity. The intestinal epithelial cells obtained at NRIAP were subjected to immunofluorescence analysis to confirm their characteristics and the presence of specific cellular markers for the enterocyte cell line (cytokeratin 18 [CK18] and sucrose-isomaltase [SI]). Additionally, using a magnetic cell sorter, cells expressing the presence of the EpCAM protein on their surface were separated. The validated cells were subsequently used for further analyses.

### 2.9. Cell Viability/Cytotoxicity Assay

The viability of porcine enterocytes treated with the obtained clove bud extracts was determined by a LIVE/DEAD™ Viability/Cytotoxicity Assay Kit (Green/Deep Red) (Invitrogen/Life Technologies Corp, Carlsbad, CA, USA) in accordance with the manufacturer’s instructions. The cells were dissociated with a 0.25% Trypsin–EDTA solution. Subsequently, cell suspensions were adjusted to a density of 1 × 10^5^ cells/mL by counting with a hemocytometer and plated on a 96-well plates. The cells were incubated overnight at 37 °C in a humidified atmosphere containing 5% CO_2_. On the next day, the obtained clove bud extracts were added to the cell cultures at the following concentrations: 20, 40, 80, 120, 160, 200, 300, 400, 600, 800, 1000, and 1200 mg/L. Untreated cells were used as controls and cultivated under the same conditions. Each concentration of the extracts was tested in six replicates. To assess the effect of the solvent (70% ethanol), cells cultured with the addition of 6% ethanol (corresponding to ethanol concentration in the medium with the highest concentration of clove extract in the culture (1200 mg/L) and 1% ethanol also were analyzed. After culturing for 6 and 24 h in the presence of the extracts, the cells were washed with PBS, and 100 μL of working solution was added to each well. The cell cultures were then incubated in the reaction mixture for 30 min at room temperature. In live cells, nonfluorescent calcein is enzymatically converted to fluorescent calcein, which is retained within cells and produces an intense uniform green fluorescence (Ex/Em: ~495 nm/~515 nm). In contrast, nuclei of dead cells or cells with a compromised plasma membrane exhibit a bright deep red/far red fluorescence (Ex/Em: maxima 660 nm/682 nm). A Synergy H1 microplate reader (BioTek/Agilent Technologies, VT, USA) was utilized to measure the absorbance of the samples. The viability of the treated cell cultures was expressed as a percentage of the respective untreated control.

### 2.10. Detection of ROS Production in Enterocytes

Trypsinized cells were seeded onto 96-well plates at the density of 5 × 10^4^ cells per well (100 µL/well) and grown overnight. The cells were then cultured for 6 and 24 h in the presence of the obtained clove extracts at concentrations ranging from 20 to 1200 mg/L. Subsequently, the cells were washed in PBS and treated with 100 µL of 2 mM H2DCF-DA (Sigma-Aldrich) at 37 °C for 30 min. In each experiment, the cells were incubated for 30 min with 2 mM tert-butyl hydroperoxide (tBHP) (Sigma-Aldrich) as an antioxidant positive control [39]. The absorbance was measured with a Synergy H1 microplate reader (Biotek) in the fluorescence mode at the following wavelengths: excitation: 492–495 nm/emission: 517–527 nm.

### 2.11. Transepithelial Electrical Resistance (TEER) Measurement

TEER of cell monolayers grown on hanging cell culture inserts (0.4 μm pore size, clear PET membrane, 6.5 mm diameter) was measured using a Millicell^®^ ERS 3.0 digital voltohmmeter with Millicell^®^ ERS 3.0 standard electrode (Millipore, Merck KGaA, CA, USA). TEER value (expressed as Ohms/cm^2^) was calculated by subtracting the background value. Cell monolayers were treated with the obtained clove extracts at concentrations ranging from 20 to 1200 mg/L. TEER was recorded after 6 and 24 h of exposure, and the data were expressed as mean of ∆Ω ± SEM of at least five independent experiments.

### 2.12. Statistical Analyses

For each variable analyzed, the assumptions for the analysis of variance were preliminarily verified. Normality of distribution was assessed using the Shapiro–Wilk test, while homogeneity of variance was checked using Levene’s test. Standard ANOVA was employed when assumptions were met; otherwise, appropriate alternative methods accounting for heterogeneity of variance were used.

A corrected *p*-value < 0.05 was considered statistically significant. All statistical analyses were performed using Jamovi software (version 2.6.44).

## 3. Results and Discussion

The objective of the present study was to determine the influence of different variables (time, amplitude, solvent/sample ratio, and temperature) on process parameters (extract yield, TPC, rhamnetin content, and antioxidant activity: FRAP, ABTS, and DPPH) utilized for extracting rhamnetin from clove buds using UAE with ethanol solvent. In accordance with the established research protocol, dried clove buds (*S. aromaticum*) were subjected to UAE with ethanol as a solvent, and the effect of different ultrasound parameters, including power, amplitude, time, solvent/sample ratio, and varying solvent temperatures, on rhamnetin yield and content, flavonoid content, and antioxidant capacity of the obtained extracts were evaluated.

Soxhlet extraction, hydrodistillation, and maceration are the commonly used techniques for extracting bioactive compounds from plant materials. Alternative methods for obtaining these compounds include microwave-assisted extraction, supercritical liquid CO_2_ extraction, and UAE [40,41]. Ethanol-based extraction is an established green chemistry technique. Ethanol is a promising solvent for extracting bioactive compounds and offers several advantages. It is also considered an environmentally friendly solvent because of the following properties: renewable production through biotechnological processes, nontoxicity, and low flammability; thus, it has a limited impact on the environment. Based on these characteristics, ethanol is considered to be more advantageous in terms of environmental protection and safety compared to other organic solvents [42,43,44]. Moreover, extracts obtained using this method do not require the application of commonly used solvents, including methanol, acetone, hexane, petroleum ether, and chloroform, which are highly toxic. Consequently, the obtained raw materials can be used as food additives and in pharmaceuticals and cosmetology products [45,46]. The efficiency of extracting bioactive compounds from plants using organic solvents can be increased by applying ultrasound. Ultrasound accelerates the extraction process and reduces energy consumption. Moreover, unlike conventional methods, UAE allows low temperatures to be used during the extraction process, enabling the protection of heat-sensitive compounds [47,48]. Previous studies have reported very high TPC values for clove extracts, although these values vary depending on the extraction method and the solvent used [49,50].

The extraction yields obtained using ultrasound and details of the experimental conditions, such as temperature, time, amplitude, and solvent/sample ratio, are presented in Table 1. The extraction conditions significantly influenced the polyphenol content. The applied extraction parameters, such as amplitude/power (90 W/mL/μm), time (20 min), and extraction liquid temperature (−30 °C), yielded a significant higher TPC value (241.03 mg GAE/100 g DM) (sample No. 6) compared to the sample extracted for 10 min at 0 °C (TPC: 135.96 mg GAE/100 g DM) (sample No. 1) (*p* < 0.004). The obtained results clearly indicate that flavonoid extraction efficiency is significantly influenced by extraction time and temperature.

Pujiarti et al. (2020) [49] and Raina et al. (2001) [50] evaluated the yield of clove leaf extracts using the hydrodistillation method and obtained yields of 2.67% and 4.8%, respectively; these yields were lower compared to those obtained in the present study: 11.99% under the following extraction conditions: 10 min and 0 °C. The extraction yield significantly increased to 21.80% after the UAE time was extended to 20 min, the extraction liquid temperature was maintained at −30 °C, and sonication conditions were applied (frequency/amplitude: 30 W/mL, 90 μm). This result confirmed that using ultrasound waves enabled a significant increase in extraction yield compared to conventional methods. Soxhlet extraction of active compounds from cloves with methanol as a solvent provided an extraction yield of 12% [51]. Frohlich et al. (2022) [30] used the maceration technique for clove bud extraction with 70% ethanol for 25 min; the authors found that the sonication method led to a higher extraction yield (14.63%) than that achieved with the maceration method (4.56%).

In the study of Fauzya et al. (2019) [26], ethanol extraction (with 70% ethanol) from clove leaves was performed using maceration for 72 h with solvent renewal, which provided an extraction yield of 12.9%. Pujiarti et al. (2020) [49] using the conventional hydrodistillation method for clove leaves, obtained extraction yields of 2.67% and 4.80%, respectively. UAE represents a sustainable alternative to classical extraction methods. UAE offers advantages in terms of equipment cost, energy consumption, and processing time; moreover, it involves the use of “green solvents” and the isolation of bioactive compounds under atmospheric pressure [52].

Ultrasound application is relatively simple and flexible and requires lower equipment costs compared to other extraction techniques. Therefore, this technique is labeled as a “green” method with a low negative environmental impact. UAE allows the use of various solvents such as water or organic solvents [53]. According to green chemistry principles, water or ethanol–water mixtures are used as solvents for extracting polar compounds, whereas oils are utilized for extracting non-polar compounds [53]. Ethanol (EtOH) is one of the most suitable solvents for green extraction. EtOH is also cheaper and less toxic than other organic solvents, which favors its application for extracting large quantities of natural products [54].

Rhamnetin and isorhamnetin were determined in the obtained extracts using the RP-UHPLC-ESI-MS method (Table 2 and Table 3). The contents of rhamnetin and isorhamnetin were substantially affected by extraction time, low temperature, and amplitude. For example, under the conditions of sonication for 20 min at −30 °C with a power/amplitude of 30 W/mL/90 μm, rhamnetin and isorhamnetin contents were 241.03 and 32.86 ppm, respectively. In comparison, rhamnetin content was 5-fold lower (46.33 ppm) and isorhamnetin content was 15.80 ppm for the sample subjected to sonication for 10 min at 0 °C.

The obtained results are consistent with those reported by Fathoni (2017) [55]. The high content of phenolic compounds in this clove leaf extract was attributed to its strong antioxidant activity, as demonstrated by in vitro assays (DPPH, FRAP, and ABTS assays). Moreover, because of this high antioxidant activity, the clove leaf extract also exhibited a strong free radical scavenging ability, which may contribute to the neutralization of toxic ROS. This result is consistent with several studies, which demonstrated that the high TPC of plant extracts obtained from *Castanea sativa* Mill. and *Commiphora mollis* (Oliv.) Engl. contributed to strong antioxidant activity measured using the same methods, i.e., DPPH and ABTS radical scavenging assays [56,57]. The results presented in Table 4 confirmed that all samples exhibited antioxidant activity. FRAP values ranged from 170.98 to 466.27 mg Trolox/100 g and differed significantly (*p* < 0.0012) among the experimental conditions. In FRAP assay, which measures the reduction of ferric ions (Fe^3+^) to ferrous ions (Fe^2+^), the values typically range from 20 to 440 μmol antioxidant equivalents per gram of extract. In the present study, these values were expressed as Trolox equivalents and were consistent with the range of reducing activity reported by Benzie and Strain (1996) [34].

In the present study, clove extracts obtained by UAE exhibited varying DPPH radical scavenging activity, ranging from 66.47% ± 0.21% to 97.28% ± 0.39% after 20 min of ultrasound extraction at 30 °C. Shende (2024) [58] found that the DPPH radical scavenging activity of steam-distilled clove extract reached 88.93% ± 0.23%.

The results of the present study suggest a clear relationship between phenolic content and antioxidant activity, as the higher DPPH, ABTS, and FRAP radical scavenging activities of the clove extracts obtained by UAE may be associated with their higher phenolic content. Another noteworthy observation is that ethanol extraction was suitable for isolating these chemical compounds exhibiting antioxidant activity. Previous studies have also used ethanol as a solvent for extracting bioactive compounds from various vegetables reported to possess strong antioxidant properties [59].

**Figure 1:**
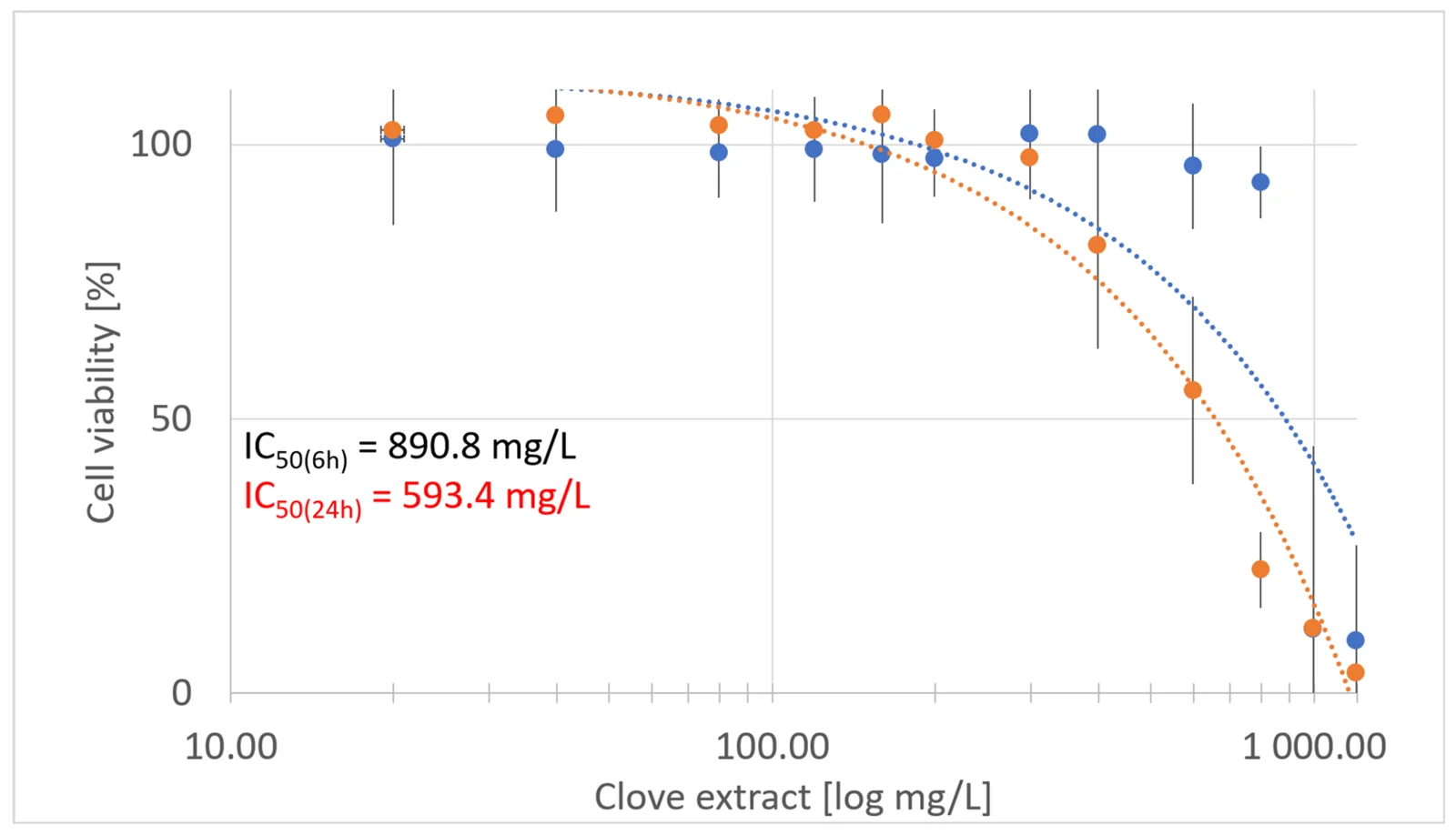
Graph of percentage of viable porcine enterocytes versus value of clove extract concentration (blue graph—6-h exposure, orange graph—24-h exposure).

Figure 1 presents the relationship between the percentage of viable porcine enterocytes and the value of clove extract concentration. The obtained clove extract (Sample No. 6) was subjected to in vitro cytotoxicity evaluation by cell culture assay. Compared to the control group, cells exposed to extracts at 1200 and 1000 mg/L concentrations exhibited a significant decrease in cell viability (*p* < 0.002 and *p* < 0.001, respectively). The results were almost the same for cells exposed for 6 h; significantly lower cell viability was observed in cells treated with 1200 and 1000 mg/L clove extract (*p* < 0.001).

After a 6-h exposure to the extracts, cytotoxicity was significantly increased in cells treated with the two highest extract concentrations (*p* < 0.05). In contrast, after 24-h exposure, cytotoxicity increased significantly only at the highest extract concentration (*p* < 0.001). Additionally, 1% and 6% of the solvent (70% ethanol) used for extraction did not exert cytotoxic effects on cell viability. The IC_50_ values were 890.8 and 593.4 mg/L for 6 and 24 h exposure, respectively.

Analyses conducted on hepatocytes (HepG2) using clove extracts showed that the IC_50_ value for the extract tested, following a 24-h incubation, exceeded 500 mg/L [60]. These results are consistent with those obtained in our own studies. In contrast, the study by Liu et al. (2014) [61] reported IC_50_ values for ethanolic clove extract ranging from 196.1 to 455 mg/L, depending on the cell line used. However, it should be noted that the cell lines examined by Liu et al. (2014) [61] were cancer cell lines.

**Figure 2:**
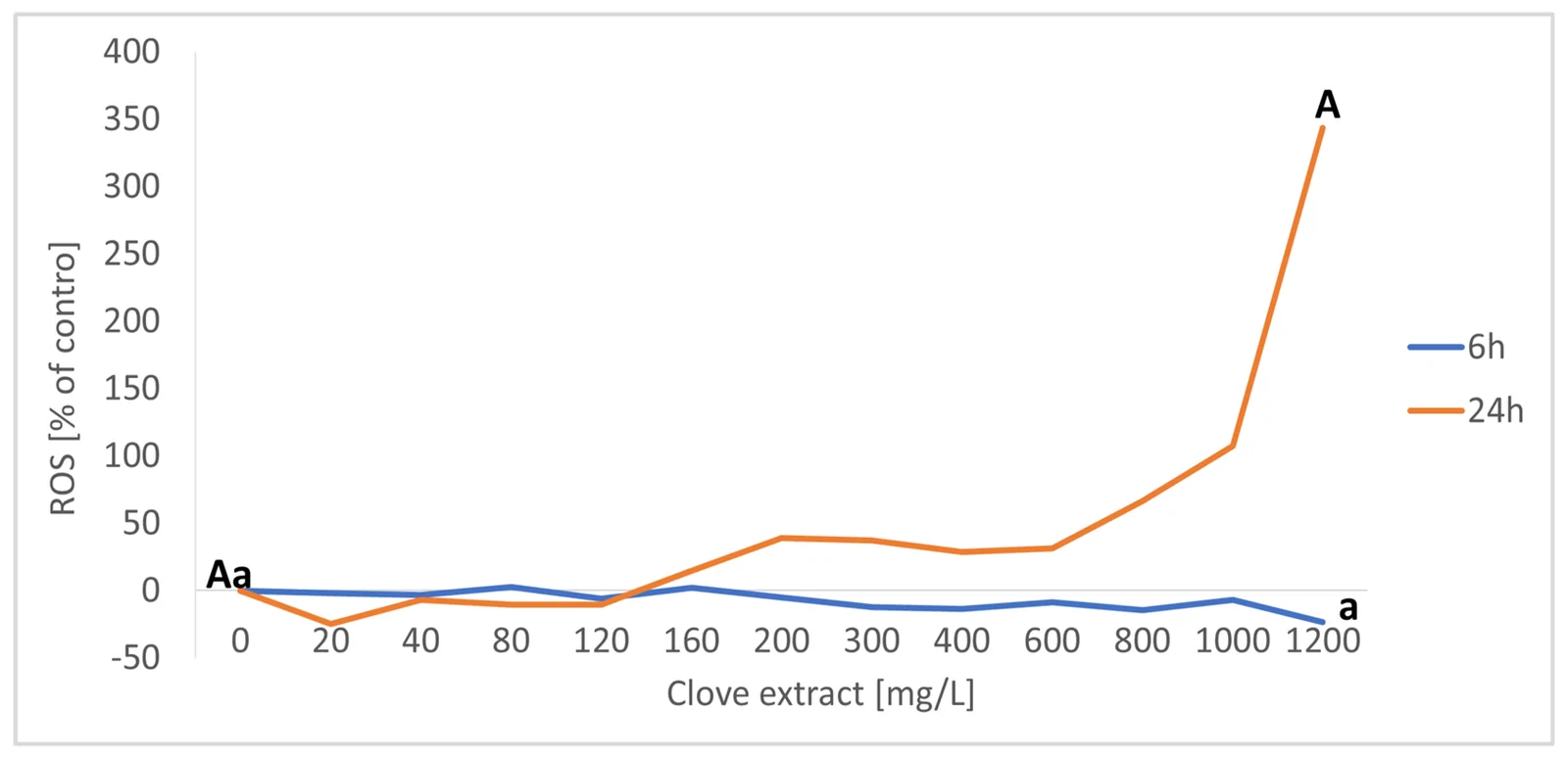
Effects of different concentrations of clove extract on ROS production in porcine enterocytes. Blue graph—6-h exposure, orange graph—24-h exposure. The same capital letter (A) indicates highly significant differences (*p* ≤ 0.001). The same small letter (a) indicates statistically significant differences (*p* ≤ 0.05); one-way ANOVA followed by Tukey’s post hoc test.

Interestingly, during the 6-h exposure, the highest clove extract concentration exhibited antioxidant effects and significantly reduced ROS levels (*p* < 0.05); in contrast, during the 24-h exposure, an opposite effect was noted, with a significant increase in ROS levels (*p* < 0.001). No significant differences were found in the other study groups. Moreover, ROS levels remained unaffected in cells cultured with 1% and 6% of the solvent (70% ethanol) used for extraction (Figure 2).

Tariq et al. (2025) [62] confirmed that clove extract can drastically increase ROS levels after 24 h of treatment, which represents one of the key mechanisms leading to cancer cell death through apoptosis induction and cell proliferation inhibition. Clove extract also exhibits an effective ability to scavenge singlet oxygen radicals, as confirmed by Sueishi and Nii (2019) [63]. Previous studies have shown that rhamnetin limits the formation of reactive oxygen species in rat cardiomyoblastic cells treated with miconazole [64]. Additionally, it is known that ethanol extract of cloves reduces the number of reactive oxygen species and protects cells against oxidative stress of various cell lines [65,66,67].

Our results indicate that short exposure (6 h) of cells to the extract, even at high concentrations, is temporally associated with reduced ROS levels. In the case of 24-h exposure, marked reductions in cell viability and barrier integrity (TEER) occur at concentrations exceeding 400–600 mg/L (above the IC_50_ value). While these findings demonstrate concentration- and time-dependent associations between decreased cell viability and elevated ROS generation, establishing direct causal mechanisms requires more targeted pathway analyses. It can be concluded that cell mortality after 24 h of exposure to higher extract concentrations may be largely the result of oxidative stress.

**Figure 3:**
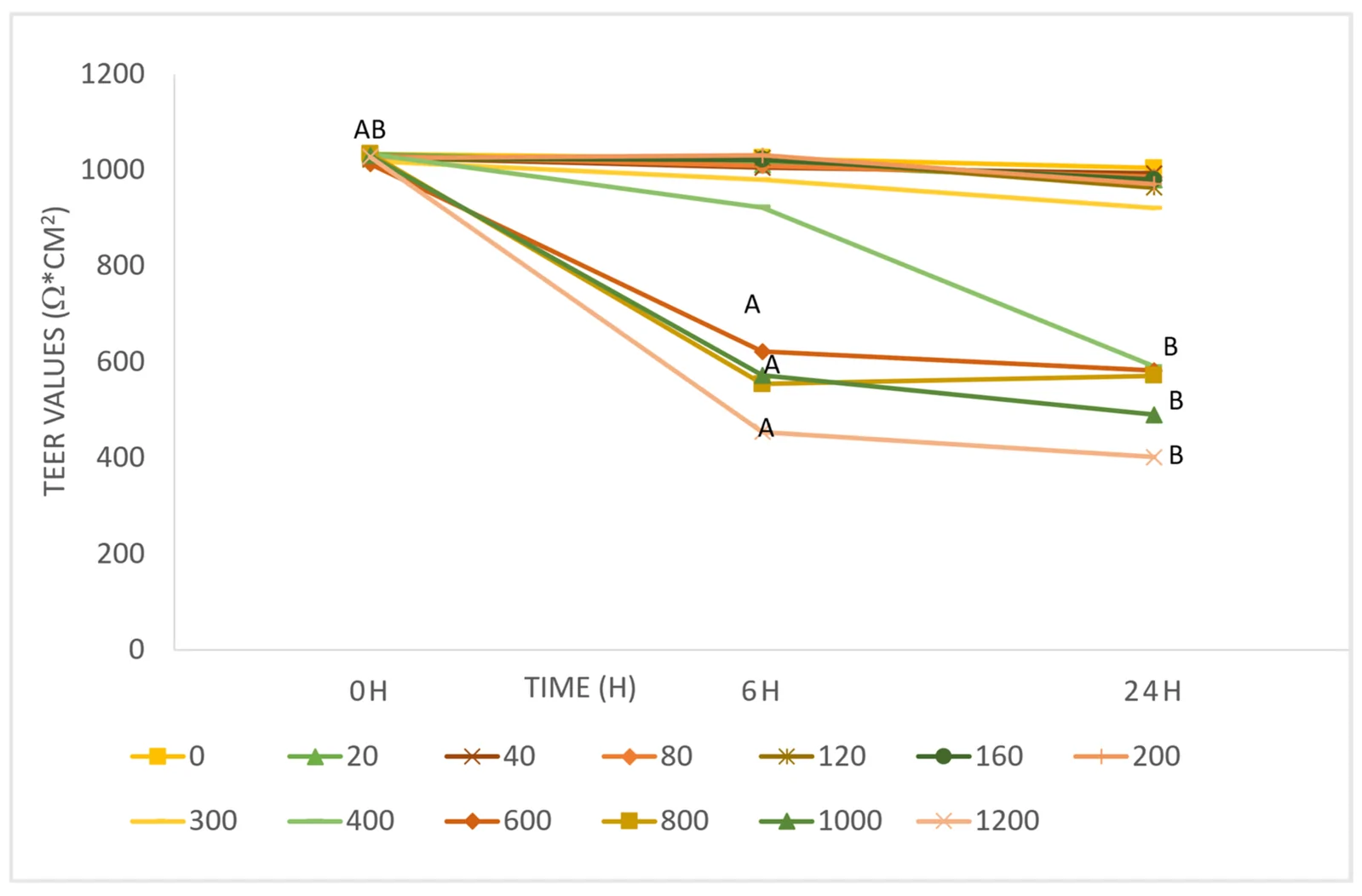
Transepithelial electrical resistance (TEER) in enterocytes after treatment with the clove extract. TEER was recorded at 6 and 24 h after extract exposure. The same capital letter (A,B) indicates highly significant differences (*p* ≤ 0.005); one-way ANOVA followed by Tukey’s post hoc test. 0-control group; 20–1200 concentrations of the clove extracts (20–1200 mg/L).

A decrease in TEER indicates impaired epithelial cell integrity (Poeta et al. 2023) [68]. In the present study, the obtained clove extract did not adversely affect TEER up to 300 mg/L concentration and did not induce a decrease in TEER over 24 h of exposure. Highly significant differences (*p* < 0.005) were observed when cells were incubated with 400 mg/L of the obtained clove extract for 24 h. However, no changes in TEER were noted after 6 h of incubation at this concentration. Extract concentrations above 400 mg/L significantly reduced the TEER value even after 6 h of incubation (*p* < 0.005) (Figure 3). Prior research indicates only that eugenol—the primary component of cloves—demonstrated properties that stabilize cell barrier integrity in IPEC-J2 cells subjected to lipopolysaccharide-induced inflammation [69]. Our analyses revealed that the extract’s effect on intestinal epithelial integrity depends on both dosage and exposure time. Concentrations lower than the corresponding IC_50_ values compromise this integrity.

## 4. Conclusions and Summary

The present study confirmed that UAE using ethanol is a highly efficient and environmentally friendly method to obtain bioactive compounds from clove buds (*S. aromaticum*). Optimization of the process parameters (20 min, 90 μm, and −30 °C) provided the highest extraction yield (21.80%) with a significantly high content of polyphenols (320.8 mg GAE/100 g DM).

The chemical profile of the extracts was specifically investigated, including the determination of rhamnetin and isorhamnetin contents using the RP-UHPLC-ESI-MS method. The results clearly indicate that sonication parameters substantially influence the isolation of these flavonoids. The application of optimal process conditions yielded rhamnetin and isorhamnetin contents of 241.03 and 32.86 ppm, respectively, representing an approximately 5-fold higher rhamnetin content compared to that achieved for samples extracted under milder conditions (10 min and 0 °C). The high content of these specific phenolic compounds provides a direct explanation for the strong antioxidant activity of the extracts, as confirmed by FRAP, ABTS, and DPPH assays.

In conclusion, the UAE technique represents an efficient alternative to conventional methods, enabling the precise isolation of rhamnetin and other bioactive compounds. The obtained results provide important guidelines for the pharmaceutical and food industries: because of their effects on cellular physiology, the high content of flavonoids requires rigorous dosage control.

In vitro studies on porcine enterocytes showed concentration- and time-dependent associations between the extract treatments and cellular parameters:

1. At low concentrations (up to 300 mg/L), the extract did not disrupt epithelial barrier integrity (no decrease in TEER) and corresponded with reduced ROS levels after 6 h of exposure.
2. At concentrations exceeding the threshold of 400 mg/L, the extract impaired epithelial barrier, decreased cell viability, and coincided with increased ROS levels after 24 h of exposure, as indicated by IC_50_ values (890.8 and 593.4 mg/L after 6 and 24 h of exposure). These associations point to potential multi-factorial responses, and further mechanistic studies are required to confirm the precise pathways involved.

Further studies should focus on discovering and validating the mechanisms underlying the effects of these extracts in more complex in vivo models, with particular emphasis on the role of rhamnetin in intestinal barrier protection.

## Figures and Tables

**Table 1:** Extraction conditions for extracting bioactive substances from clove buds (*Syzygium aromaticum*).

Sample Name ^1^	Extraction Conditions				
	Extraction Time (min)	Extraction Temperature (°C)	Liquid-to-Solid Ratio (mg/mL)	Frequency/Amplitude W/mL/μm	Ethanol Concentration (%)
Sample 1	10	0	112	30:90	70
Sample 2	20	0	112	30:90	70
Sample 3	10	−10	112	30:90	70
Sample 4	20	−10	112	30:90	70
Sample 5	10	−30	112	30:90	70
Sample 6	20	−30	112	30:90	70

^1^: Each variant was analyzed in three replicates.

**Table 2:** Rhamnetin and isorhamnetin content (ppm) in ethanolic clove extracts determined by reversed-phase ultra-high-performance liquid chromatography coupled with electrospray ionization mass spectrometry.

Clove Extracts/Sample	Rhamnetin *	Isorhamnetin **
Sample 1	46.33 ± 1.85 ^b^	15.80 ± 0.50 ^b^
Sample 2	44.61 ± 1.90 ^b^	16.50 ± 0.48 ^b^
Sample 3	48.53 ± 2.10 ^b^	15.50 ± 0.42 ^b^
Sample 4	46.49 ± 2.01 ^b^	15.93 ± 0.51 ^b^
Sample 5	224.23 ± 3.42 ^a^	25.65 ± 0.86 ^a^
Sample 6	241.03 ± 3.62 ^a^	32.86 ± 0.94 ^a^
RSD %	4.23	3.22
*p*-value	0.004	0.012

* measurement was performed after a 10-fold dilution of the sample in methanol. Different superscript letters in each column indicate significant differences (*p* < 0.05). ** isorhamnetin expressed as rhamnetin. Different superscript letters in each column indicate significant differences (*p* < 0.05).

**Table 3:** Parameters and standard calibration curve plot.

Target Cmpd Name	Rhamnetin
Target Cmpd Chromatogram	EIC 315.0510 ± 0.01 ± All
Target Cmpd RT window	13.0 ± 0.2 min
Calib Curve Fit	Quadratic
Calib Curve Origin	Exclude
Calib Curve Weighting	None
Calib Curve Equation	y = −44,925.565182 x^2^ + 1,582,231.409507 x + −192,617.984685
Calib Curve Correlation Coefficient	0.9998

**Table 4:** TPC and antioxidant properties of clove extracts expressed as mean activity (±SD): TPC, DPPH, RSA, FRAP, and ABTS.

Clove Extracts/Sample	TPC [mg GAE/ 100 g DM]	FRAP [mg Trolox/100 g]	ABTS [μM Trolox/100 g]	DPPH [%] RSA	Extraction Yield [%]
Sample 1	135.96 ± 1.75 ^d^	170.98 ± 4.50 ^e^	8.78 ± 0.70 ^c^	66.47 ± 0.21 ^d^	11.99
Sample 2	147.61 ± 1.80 ^cd^	244.25 ± 4.88 ^d^	9.53 ± 0.78 ^bc^	72.16 ± 0.24 ^d^	13.29
Sample 3	159.27 ± 1.88 ^c^	321.39 ± 6.44 ^c^	10.28 ± 0.88 ^bc^	77.86 ± 0.29 ^c^	14.38
Sample 4	168.98 ± 1.95 ^c^	391.94 ± 7.89 ^b^	10.91 ± 0.92 ^ab^	82.61 ± 0.32 ^c^	15.69
Sample 5	226.23 ± 2.08 ^b^	450.50 ± 9.18 ^a^	12.54 ± 0.99 ^a^	90.95 ± 0.33 ^b^	20.05
Sample 6	320.08 ± 2.40 ^a^	466.27 ± 9.38 ^a^	12.98 ± 1.04 ^a^	97.27 ± 0.39 ^a^	21.80
*p*-value	0.004	0.0012	0.056	0.001	

Different superscript letters in each column indicate significant differences (*p* < 0.05).

## Data Availability

The data and models used in this study are archived in the Repository of the National Research Institute of Animal Production and are available from the corresponding author upon reasonable request.

## Abbreviations

The following abbreviations are used in this manuscript:

UAE	Ultrasound-Assisted Extraction
TPC	Total Phenolic Content ROS
TEER	Transepithelial Electrical Resistance
DPPH	2,2-diphenyl-1-picrylhydrazyl
ABTS	2,2′-azino-bis(3-ethylbenzothiazoline-6-sulfonic acid)
FRAP	Ferric Reducing Antioxidant Power
DM	Dry Matter
DW	Dry Weight
GAE	Gallic Acid Equivalent
TE	Trolox Equivalent
RP-UHPLC-ESI-MS	Reversed-Phase Ultra-High-Performance Liquid Chromatography Coupled with Electrospray Ionization Mass Spectrometry
RSA	Radical Scavenging Activity
IC_50_	Half Maximal Inhibitory Concentration
PBS	Phosphate-Buffered Saline
EDTA	Ethylenediaminetetraacetic Acid
ROCK	Rho Kinase
DMEM/F12	Dulbecco’s Modified Eagle Medium/Nutrient Mixture F-12
FBS	Fetal Bovine Serum
ITS	Insulin-Transferrin-Selenium
AAS	Antibiotic Antimycotic Solution
EGF	Epidermal Growth Factor
CK18	Cytokeratin 18
SI	Sucrase-Isomaltase
EpCAM	Epithelial Cell Adhesion Molecule
tBHP	Tert-Butyl Hydroperoxide
SEM	Standard Error of the Mean
SD	Standard Deviation
RSD	Relative Standard Deviation
ANOVA	Analysis of Variance
EtOH	Ethanol
CO_2_	Carbon Dioxide
